# Tissue Antioxidant Status and Lipid Peroxidation Are Related to Dietary Intake of n-3 Polyunsaturated Acids: A Rabbit Model

**DOI:** 10.3390/antiox10050681

**Published:** 2021-04-27

**Authors:** Simona Mattioli, Giulia Collodel, Cinzia Signorini, Elisa Cotozzolo, Daria Noto, Daniela Cerretani, Lucia Micheli, Anna Ida Fiaschi, Gabriele Brecchia, Laura Menchetti, Elena Moretti, Camille Oger, Claudio De Felice, Cesare Castellini

**Affiliations:** 1 Department of Agricultural, Environmental and Food Science, University of Perugia, Borgo 20 Giugno, 74, 06123 Perugia, Italy; simona.mattioli@unipg.it (S.M.); elisa.cotozzolo@student.unipg.it (E.C.); cesare.castellini@unipg.it (C.C.); 2 Department of Molecular and Developmental Medicine, University of Siena, Policlinico Santa Maria alle Scotte, Viale Bracci 16, 53100 Siena, Italy; cinzia.signorini@unisi.it (C.S.); noto@student.unisi.it (D.N.); elena.moretti@unisi.it (E.M.); 3 Department of Medicine, Surgery and Neurosciences, University of Siena, Policlinico Santa Maria alle Scotte, Viale Bracci, 16, 53100 Siena, Italy; daniela.cerretani@unisi.it (D.C.); lucia.micheli@unisi.it (L.M.); annaida.fiaschi@unisi.it (A.I.F.); 4 Department of Veterinary Medicine, University of Milan, Via dell’Università 6, 26900 Lodi, Italy; gabriele.brecchia@unimi.it; 5 Department of Agricultural and Agri-Food Sciences and Technologies, University of Bologna, Viale Fanin 46, 40138 Bologna, Italy; laura.menchetti7@gmail.com; 6 Institut des Biomolécules Max Mousseron (IBMM), UMR, CNRS, Université de Montpellier, ENSCM, 5247 Montpellier, France; camille.oger@umontpellier.fr; 7 Neonatal Intensive Care Unit, Azienda Ospedaliera Universitaria Senese, 53100 Siena, Italy; c.defelice@ao-siena.toscana.it

**Keywords:** brain, cellular antioxidants, isoprostanoids, liver, polyunsaturated fatty acids, rabbit, testis, vitamin E

## Abstract

Polyunsaturated fatty acid (PUFA) metabolism and tissue distribution is modulated by the oxidation of these molecules. This research aimed to investigate the implication of dietary n-3 PUFA supplementation (precursor and long-chain PUFA) on the PUFA profile and oxidative status of the liver, testis, and brain of adult rabbit bucks. Twenty New Zealand White rabbit bucks were divided into four experimental groups (*n* = 5 per group) and were fed different diets for 110 days: control (CNT), standard diet containing 50 mg/kg alpha-tocopheryl acetate (vitamin E); CNT+, standard diet + 200 mg/kg vitamin E; FLAX, standard diet + 10% flaxseed + 200 mg/kg vitamin E; or FISH, standard diet + 3.5% fish oil + 200 mg/kg vitamin E. Antioxidants (enzymatic and non-enzymatic), oxidative status (malondialdehyde and isoprostanoids), and n-3 and n-6 PUFAs of tissues were analysed. A chain mechanism of oxidant/antioxidant molecules, which largely depended on the particular PUFA composition, was delineated in the different organs. The liver showed an oxidant/antioxidant profile and lipid pathways widely modulated by PUFA and vitamin E administration; on the other hand, the testis’ oxidative profile rather than its lipid profile seemed to be particularly affected, an outcome opposite to that of the brain (modulation operated by dietary PUFA).

## 1. Introduction

The role of polyunsaturated fatty acids (PUFAs), especially the n-3 series, on human health is well known. In particular, eicosapentaenoic (EPA, 20:5n-3) and docosahexaenoic (DHA,22:6n-3) acids, the long-chain polyunsaturated fatty acid derivatives (LCPs) of alpha-linolenic acid (ALA, 18:3n-3), positively modulate several physiological processes, including the regulation of plasma lipid levels [1,2], cardiovascular [3,4] and immune function [5], glucose metabolism [6], neuronal development, visual activity [7], and male and female fertility [8,9].

Saturated fatty acids (SFAs) and monounsaturated fatty acids (MUFAs) can be synthesised de novo, whereas essential PUFAs (LA and ALA) must be obtained via dietary intake. Epidemiological studies have underlined the importance of a balanced intake of n-6 and n-3 PUFAs [10,11]: recent recommendations for human diets [12,13] suggest increasing n-3 PUFA consumption and decreasing the n-6:n-3 PUFA ratio from 10–20:1 (the typical ratio in Western diets) to 4:1.

To promote higher intake of n-3 PUFAs, two main strategies could be used: increase the consumption of n-3 precursors through vegetables or seeds containing ALA, or the direct intake of n-3 PUFAs contained in fish and other marine products (algae) rich in LCPs. However, the two dietary strategies are not equally effective. Indeed, dietary PUFAs (ALA or LCP) are incorporated into the body with different patterns and efficiencies [14]. For example, the genetic background (related to species, animal breed, gender, etc. [15]) and the gut microenvironment [16,17] largely influence dietary lipid absorption and metabolism. Furthermore, the competition between linolenic acid (LA, 18:2n-6) and ALA, which have their own distinct bioactivity compared to LCPs [18], plays a crucial role in the metabolic fate of dietary PUFAs. LA and ALA compete for the same desaturase and elongase enzymes, which means that their rate of conversion into arachidonic acid (ARA, 20:4n-6) and DHA, respectively, depends on the relative availability of precursors and enzymes [15]. In addition, the ingestion of PUFAs modulates membrane composition and function, eicosanoid synthesis, and signalling, as well as the regulation of gene expression [19,20,21], in an organ-dependent manner.

Castellini et al. [22] demonstrated that the dietary administration of n-3 PUFA, both as LCPs (fish oil) or precursor (flaxseed), determined an enrichment in such fatty acids (FAs) in rabbit testes. Furthermore, the same authors reported that, although the liver is the main metabolic centre for LCP synthesis, reproductive tissues (i.e., ovaries) have a noticeable role in LCP generation [23]. Similarly, the brain is very sensitive to changes in dietary fat intake but in a region-specific manner [24]. In particular, the brain of mice fed a diet containing a 7:1 LA:ALA ratio showed more sensitive, region-specific changes in dietary fat [24].

PUFA metabolism is also affected by the oxidation of such molecules. PUFAs are very susceptible to lipid peroxidation, which plays a prominent role in many acute and chronic diseases and even in the normal ageing process [25]. Increasing evidence indicates the involvement of lipid peroxidation in such disorders; thus, the biomarkers for lipid peroxidation have attracted more attention. General markers are mainly represented by malondialdehyde (MDA) or are specifically characterised by single molecules. Isoprostanoids, which are prostaglandin (PG)-like substances, are produced in vivo by free radical–induced peroxidation of FAs [26] and represent a very specific and sensitive index of lipid peroxidation. Isoprostanoids are derived from FAs esterified in cell membrane phospholipids and released in a free form in biological fluids. The main isoprostanoid marker is F_2_-isoprostane (F_2_-IsoP), which originates by non-enzyme oxidation of ARA. However, a high dietary level of n-3 PUFAs leads to the formation F_3_-IsoPs and F_4_-neuroprostane (F_4_-NeuroP) from non-enzymatic oxidisation of EPA and DHA, respectively [27].

Due to the susceptibility of PUFA to oxidation, the role of antioxidants has received extensive attention. Accordingly, antioxidant protection is always required [28] when dietary PUFAs are supplied. Some studies in rabbits [29,30,31,32] have shown an improvement in muscle oxidative stability by feeding animals with supra-nutritional levels of various antioxidants, both as synthetic additives [33] and as natural compounds [34,35]. In particular, one of the main molecules that protects PUFA is vitamin E, which is one of the most vital lipid-soluble antioxidants. It protects membranes from oxidation by reacting with lipid radicals produced in the lipid peroxidation chain reaction [36]. The hydrophobic nature of vitamin E makes it preferentially located in fat-storage organs, fat deposits, and cell membranes. Vitamin E is transported around the body as an element of plasma lipoproteins and then transferred from the plasma to cells through uptake facilitated by receptor-mediated lipoprotein endocytosis, lipid transfer proteins and lipases, and selective lipid uptake [37]. The body’s vitamin E levels change in view of many different conditions (dietary intake, metabolic uptake, stress, selenium content, etc.), which are currently considered for health and welfare assessment [38]. As an antioxidant, vitamin E works in synergy with an endogen antioxidant system to scavenge free radicals. This system includes ascorbic acid and thiol antioxidants (e.g., glutathione).

To the best of our knowledge, this is the first study that evaluated the implication of n-3 PUFA dietary supplementation (precursor and LCPs) on the PUFA profile and oxidative status of specific rabbit organs. The lipid oxidative status, isoprostanoid generation, and enzymatic/non-enzymatic antioxidant content were evaluated in the liver, brain, and testis of rabbit bucks fed flaxseed- or fish-oil-enriched diets.

## 2. Materials and Methods

### 2.1. Animals and Experimental Design

Twenty New Zealand White rabbit bucks, 140 days old, were selected and divided into four experimental groups (*n* = 5 per group). Each group was fed a different diet (Table 1).

- The negative control (CNT−) group was fed ad libitum a standard diet containing 50 mg/kg alpha-tocopheryl acetate (vitamin–mineral premix).
- The positive control (CNT+) group was fed ad libitum a standard diet that also included 200 mg/kg alpha-tocopheryl acetate (50 mg/kg contained in the vitamin–mineral premix + 150 mg/kg of alpha-tocopheryl acetate).
- The FLAX group was fed a standard diet supplemented with 10% extruded flaxseed and 200 mg/kg alpha-tocopheryl acetate (50 mg/kg contained in the vitamin–mineral premix + 150 mg/kg of alpha-tocopheryl acetate).
- The FO group was fed a standard diet supplemented with 3.5% fish oil (NORDIC NATURALS omega-3^®^; Watsonville, CA, USA) and 200 mg/kg alpha-tocopheryl acetate (50 mg/kg contained in the vitamin–mineral premix + 150 mg/kg of alpha-tocopheryl acetate).

The experimental protocol involved 110 days of feeding. This study was conducted in accordance with the Guiding Principles in the Use of Animals and approved by the Animal Ethics Monitoring Committee of the University of Siena (CEL AOUS; authorisation no. 265/2018-PR, ISOPRO 7DF19.23).

### 2.2. Sampling of Rabbit Organs

At the end of the experiment, the rabbits were killed in the university facility after an overdose of barbiturates as approved by the Animal Ethics Monitoring Committee of the University of Siena. Their whole liver, brain (without cerebellum), and testes (both sides) were accurately removed, and portions were placed in sterile tubes and stored at −80 °C for the evaluation of the FA profile, isoprostanoids, MDA, vitamin E, ascorbic acid (AA), catalase (CAT), glutathione peroxidase (GPX), glutathione reductase (GR), and glutathione reduced (GSH) and oxidised (GSSG). Five samples per organ/buck were collected and analysed.

### 2.3. Analytical Determinations

#### 2.3.1. Determination of the Fatty Acid Content in Rabbit Organs

Briefly, by mechanical shear using a potter homogeniser and a Teflon^®^ pestle, liver, testis, and brain tissues (2 g) were homogenised (10% *w*/*v*) in phosphate-buffered saline (PBS), pH 7.4 containing butylated hydroxytoluene (BHT) solution (0.06%). Lipids were extracted from the different finely ground tissues according to Folch et al. [39], the esterification was performed according to Christie [40]. The trans-methylation procedure was performed using eicosenoic acid methyl esters (Sigma-Aldrich, Schnelldorf, Germany) as an internal standard. The recovery rates of the internal standard were 96% ± 2%, 82% ± 5%, and 83% ± 3% in the liver, brain, and testis, respectively.

The FA composition was determined using a Varian gas chromatograph (CP-3800) equipped with a flame ionisation detector and a capillary column 100 m long × 0.25 mm × 0.2 μm film (Supelco, Bellefonte, PA, USA). Helium was used as the carrier gas with a flow of 0.6 mL/min. The split ratio was 1:20. The oven temperature was programmed as reported by Mattioli et al. [23]. Individual fatty acid methyl esters (FAMEs) were identified by comparing the relative retention times of peaks in the sample with those of a standard mixture (FAME Mix Supelco, Sigma-Aldrich). The fatty acids are expressed as a percentage of total FAs. The average amount of each FA was used to calculate the sum of the total LCPs from n-6 and n-3 series.

#### 2.3.2. Determination of the Vitamin E, Ascorbic Acid, and Catalase Levels in Rabbit Organs

Vitamin E (α-tocopherol and α-tocotrienol isoforms) was extracted from tissues according to Schüep and Rettenmaier [41] with some modifications. Briefly, the liver, testis, or brain (2 g) was homogenised with aqueous butylated hydroxytoluene (BHT) (0.06%), saponified with ethanolic potassium hydroxide (KOH, 60%) at 70 °C for 30 min and extracted three times with hexane/ethyl acetate (9:1, *v*/*v*). The extract was dried under a nitrogen gas (N_2_) stream and dissolved with acetonitrile. The high-performance liquid chromatography (HPLC) analysis of α-tocopherol and α-tocotrienol was performed using a Perkin Elmer series 200 pump, equipped with an autosampler (model AS 950-10, Tokyo, Japan) and a Synergy Hydro-RP column (4 μm, 4.6 × 100 mm; Phenomenex, Bologna, Italy). The flow rate was 2 mL/min, and tocols were identified using a fluorescence detector (model Jasco, FP-1525) set at excitation and emission wavelengths of 295 and 328 nm, respectively. Quantification was based on external calibration curves prepared with varying amounts of pure standard compounds (Sigma-Aldrich, Bornem, Belgium) in ethanol.

AA levels were measured in rabbit tissue by HPLC as described by Ross [42] with minor modifications. Rabbit tissue samples were homogenised in ethylenediaminetetraacetic acid (EDTA)-K^+^-phosphate buffer pH 7.4 (1:4, *v*/*v*) at 0 °C, and 0.6 mL aliquots of the samples were added to an equal volume of 10% (*w*/*v*) metaphosphoric acid. The samples were immediately centrifuged at 2000× *g* for 10 min at 0 °C. The supernatants were filtered (Anotop 0.2 μm; Merck, Kenilworth, NJ, USA) and 20 μL was injected into the HPLC column. AA was quantified by ultraviolet (UV) reverse-phase HPLC using a Waters 600 E System Controller HPLC equipped with a Waters Dual k 2487 detector (Milford, MA, USA) set at 262 nm. A 5 μm Ultrasphere ODS column (Beckman, San Ramon, CA, USA) was used with acetonitrile:water (49:51, *v*/*v*) as the mobile phase at a flow rate of 0.8 mL/min. AA concentrations were calculated by peak areas determined using an Agilent 3395 integrator (Agilent Technologies, Santa Clara, CA, USA); the results are expressed in nmol/g tissue.

To determine CAT activity, a microassay procedure described by Johansson and Borg [43] was used. Rabbit organs were homogenised in ice-cold phosphate buffer (0.125 M, pH 7.4) containing 1 mM EDTA and then centrifuged at 10,000× *g* for 15 min at 4 °C. This method is based on the reaction of CAT with methanol in the presence of an optimal concentration of hydrogen peroxide (H_2_O_2_). The formaldehyde production was measured at 540 nm with 4-amino-3-hydrazino-5-mercapto-1,2,4-triazole (Purpald) as a chromogen. One unit of CAT activity is defined as the amount of enzyme that will cause the formation of 1 nmol/min of formaldehyde at 25 °C. The results are expressed as U/mg protein.

#### 2.3.3. Determination of the Glutathione-Related Enzyme Activities and Glutathione Molecular Forms in Rabbit Organs

GR activity was determined by a microassay procedure described by Cribb et al. [44]. The method is based on the increase in absorbance at 415 nm when 5,5′-dithiobis(2-nitrobenzoic acid) (DTNB) is reduced by GSH generated from an excess of GSSG. Briefly, rabbit tissue samples were diluted (1:1) in cold 0.1 M phosphate buffer, pH 7.4, with 0.25 M sucrose and centrifuged at 40,000× *g* for 20 min at 4 °C. The supernatants were used for the GR assay. The rate of increase in absorption is directly proportional to the amount of GR in the sample. The results are expressed as U/mg protein.

GPX activity was determined spectrophotometrically at 340 nm as described by Flohé and Günzler [45]. Rabbit tissues were treated using the same procedure as described for GR. The GPX activity is quantitated by measuring the change in absorbance at 340 nm caused by the oxidation of NADPH to NADP. One unit of GPX activity is defined as the amount of enzyme that oxidises 1 μmol/min of NADPH at 37 °C. GPX is expressed as U/mg protein.

Total GSH and GSSG levels were evaluated in rabbit tissue using an enzymatic microassay procedure [46]. Rabbit organ samples were homogenised in EDTA-K^+^-phosphate buffer pH 7.4 (1:4, *v*/*v*) at 0 °C; 0.3 mL aliquots of the samples were added to an equal volume of 10% (*w*/*v*) metaphosphoric acid and immediately centrifuged at 2000× *g* for 10 min at 0 °C. The sulphydryl group of GSH reacts with DTNB and produces a yellow-coloured product, namely 5-thio-2-nitrobenzoic acid (TNB). The rate of TNB production is directly proportional to the concentration of GSH, based on the absorbance at 415 nm. The results are expressed as nmol GSH or GSSG/mg protein. The GSH/GSSG ratio was calculated and reported. The enzyme activities were evaluated in the cytosol.

Protein concentrations were determined by the method of Lowry et al. [47] and the calibration curves were prepared with dry bovine serum albumin.

#### 2.3.4. Determination of Malondialdehyde, F_2_-Isoprostane, F_3_-Isoprostane, and F_4_-Neuroprostane Content in Rabbit Organs

Lipid peroxidation in the organs was assessed by the MDA level. Rabbit tissue samples were homogenised in a 0.04 M K^+^-phosphate buffer (pH 7.4) containing 0.01% BHT (1:5 *w*/*v*, 0 °C) to prevent the artificial oxidation of free PUFAs during the assay. The homogenate was deproteinised with acetonitrile (1:1) and then centrifuged at 3000× *g* for 15 min. The supernatants were used for MDA analysis after pre-column derivatisation with 2,4-dinitrophenylhydrazine according to the method published by Shara et al. [48] with minor modifications. The samples were immediately stirred, extracted with 5 mL pentane, and dried using nitrogen. MDA hydrazone was quantified by isocratic HPLC using a Waters 600 E system controller HPLC instrument equipped with a Waters Dual λ 2487 UV detector set at 307 nm. A 5 μm Ultrasphere ODS C18 column was used with a mobile phase composed of acetonitrile (45%) and HCl 0.01 N (55%) at a flow rate of 0.8 mL/min. A calibration curve with concentrations of MDA ranging from 0.2 to 10 nmol/mL was used for quantification. The MDA concentration was calculated by peak areas using an Agilent 3395 integrator. The results are expressed as nmol/g tissue.

The levels of isoprostanes (free form plus esterified)—F_2_-IsoPs, F_3_-IsoPs, and F_4_-NeuroPs—were determined by gas chromatography/negative-ion chemical ionisation tandem mass spectrometry (GC/NICI-MS/MS). At the assay time, tissue samples (liver, testis, brain) were thawed and homogenised (10% *w*/*v*) in phosphate-buffered saline (PBS, pH 7.4) in the presence of BHT (100 μM prepared in absolute ethanol) and then hydrolysed by incubation (1 mL) in aqueous KOH (1 mM, 500 μL) at 45 °C for 45 min. Subsequently, samples were treated with HCl (1 mM, 500 μL) and spiked with the tetradeuterated derivative of prostaglandin F_2α_ (PGF_2α_-d_4_; 500 pg) as an internal standard. Solid-phase extraction procedures were carried out, according to a previously reported methodology [49]. Briefly, ethyl acetate (8 mL) was added to each sample to extract total lipids; the solution was centrifuged at 1000× *g* for 5 min at room temperature. The obtained lipid extract was applied to an aminopropyl (NH_2_) cartridge (500 mg Sorbent Cartridge, 55–105 μm Particle Size, 6 cc, Waters) to extract and collect isoprostanoids. All the final eluates were derivatised to convert the carboxylic group of isoprostanoids or PGF_2α_-d_4_ into pentafluorobenzyl ester and the hydroxyl group into trimethylsilyl ethers, as previously reported [50]. The derivatised isoprostanoids were detected and quantified by GC/NICI-MS/MS. The mass ions determined were the product ions at *m/z* 299 (F_2_-IsoPs), *m/z* 297 (F_3_-IsoPs), and *m/z* 323 (F_4_-NeuroPs). For the internal standard (PGF_2α_-d_4_), the mass ions determined were the product ions at *m/z* 303. All the product ions were derived from the [M-181]^-^ precursor ions. Reference molecules for F_2_-IsoPs, F_3_-IsoPs, and PGF_2α_-d_4_ were purchased (Cayman Chemical, Ann Arbor, MI, USA). 4(RS)-F_4t_-NeuroP, 10(R)−10-F_4t_-NeuroP, and 10(S)−10-F_4t_-NeuroP were synthesised by C.O. and used as reference molecules for F_4_-NeuroP determination.

### 2.4. Statistical Analysis

All the traits (vitamin E, MDA, AA, CAT, GSH, GSSG, GPX, GR, PUFA, and isoprostanoids) were analysed with a mixed model to evaluate the fixed effect of diet (CNT−, CNT+, FLAX, FO) [51] and the random effects on rabbit bucks. Least squares (LS) means and pooled standard error (SE) are reported. The Bonferroni correction was applied for multiple comparisons. The significance was set at *p* ≤ 0.05.

Two discriminant analyses (DAs), independent of the diets, were also performed to determine the combinations of variables describing antioxidants and the lipid and isoprostane profiles that best discriminated the organs, regardless of diet. The Mahalanobis distance was used to verify multivariate normality and to identify the presence of multivariate outliers, while pooled within-groups matrices were used to verify multicollinearity. Finally, Box’s M was used to verify the equality of covariance, although its significance was ignored because the results of models using within- and separate group covariances were equal. Discriminant loadings and centroids, indicating the mean discriminant scores for each organ, are reported. The importance of each discriminant function was evaluated through its relative percentage of variance and Wilks’ lambda (the smaller Wilks’ lambda, the more important the function). The performances of the final DAs were, instead, estimated by running a leave-one-out cross-validation, which calculates the probability for each sample to be accurately classified in the correct organ [52,53,54].

## 3. Results

### 3.1. Oxidative Status and Antioxidant Content of Organs

#### 3.1.1. Non-enzymatic Antioxidants

**Figure 1:**
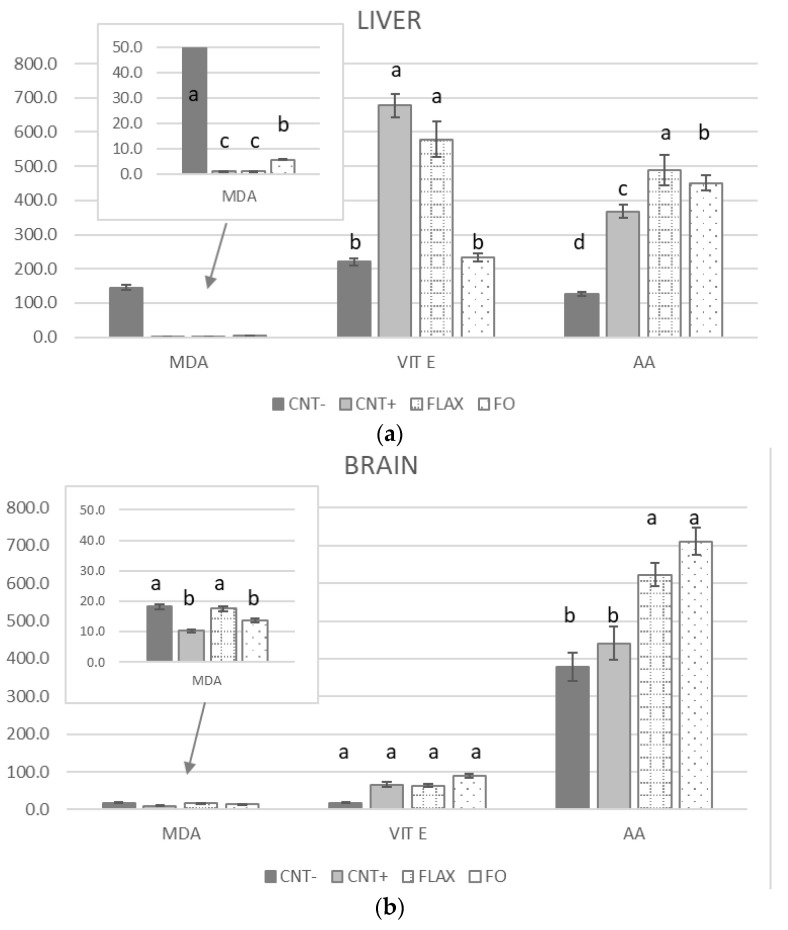

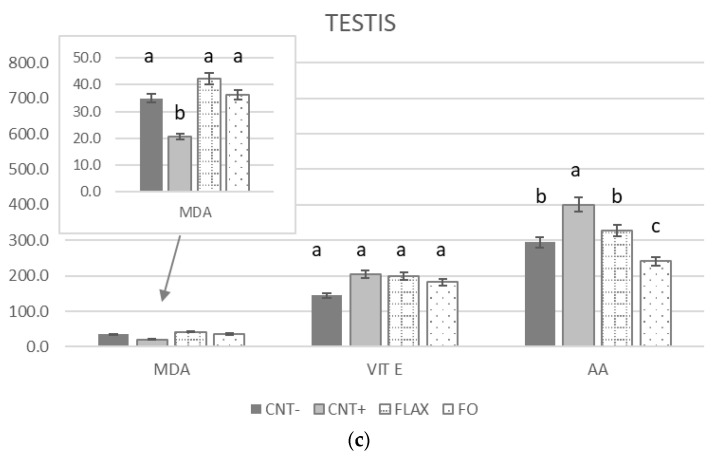
The effect of experimental diets on the oxidative status (malondialdehyde (MDA), nmol/g) and non-enzymatic antioxidant (vitamin E, ng/g, and ascorbic acid (AA), nmol/g) content of rabbit (**a**) liver, (**b**) brain, and (**c**) testis. When values differences were too wide to show a complete graph, a support window was added to the main one. The bars represent least squares means + 95% upper and lower limits. The letters a–c indicate a significant difference between dietary groups (*p* < 0.05, Bonferroni post hoc test).

Figure 1 reports the MDA content and non-enzymatic antioxidants (vitamin E and AA) of the liver, brain, and testis. In the liver (Figure 1a), the MDA content was highest in the CNT− group followed by that in the FO group. No significant differences between the FLAX and CNT+ groups were found. Conversely, vitamin E was lower in the CNT− and FO groups relative to that in the CNT+ and FLAX groups. In the brain and testis (Figure 1b,c, respectively), the MDA content was the lowest in the CNT+ group, followed by the FO in the brain. The administration of 200 mg/kg alpha-tocopheryl acetate (CNT+) simultaneously or not with n-3 PUFAs did not affect the vitamin E level in either the brain or testis. Conversely to the vitamin E level, in the liver and brain, the AA level was higher in both n-3 PUFA–supplemented groups compared with that in the CNT− or CNT+ groups. In the testis, the AA level was higher in the CNT+ groups than in the n-3 PUFA–supplemented groups and the CNT− group.

#### 3.1.2. Enzymatic Antioxidants

**Figure 2:**
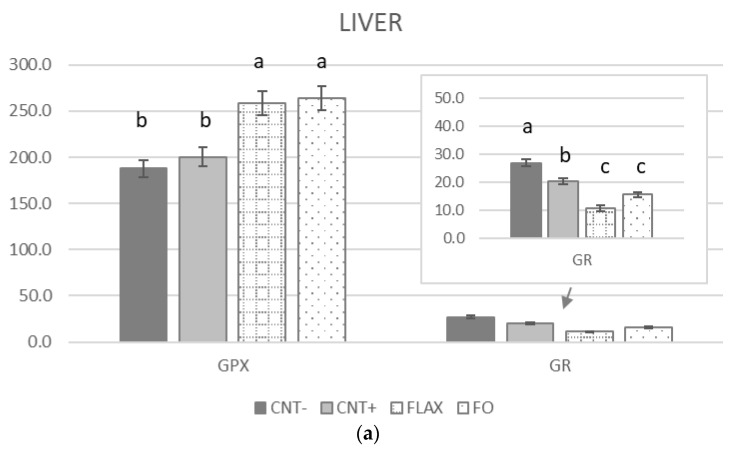

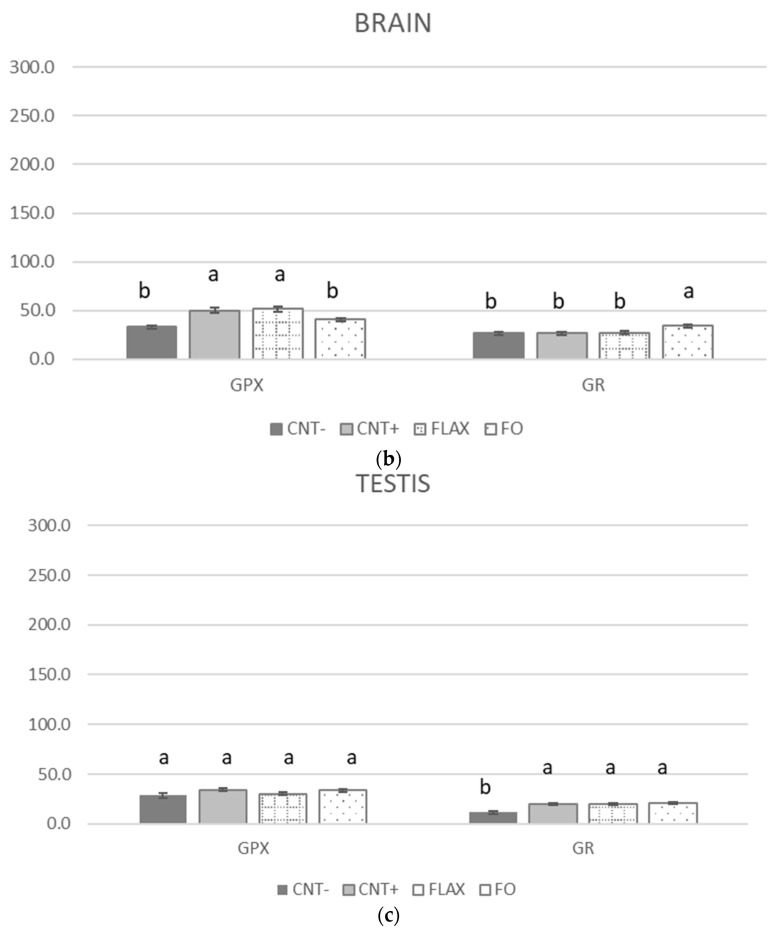
The effect of experimental diets on glutathione peroxidase (GPX) and glutathione reductase (GR) activites (U/mg protein) in rabbit (**a**) liver, (**b**) brain, and (**c**) testis. When values differences were too wide to show a complete graph, a support window was added to the main one. The bars represent least squares means + 95% upper and lower limits. The letters a–c indicate a significant difference between dietary groups (*p* < 0.05, Bonferroni post hoc test).

The GPX activity of the liver (Figure 2a) was significantly higher in the n-3 PUFA–supplemented groups compared with that in the CNT− and CNT+ groups. GR activity showed the opposite results. The brain (Figure 2b) showed higher GPX activity in the FLAX and CNT+ groups compared with that in the FO and CNT− groups. GR activity was similar in all the groups, apart from a higher value for the FO group. In the testis (Figure 2c), the GPX activity did not show significant differences, conversely to that in the brain. The GR activity was higher in all groups compared with that in the CNT− group.

**Figure 3:**
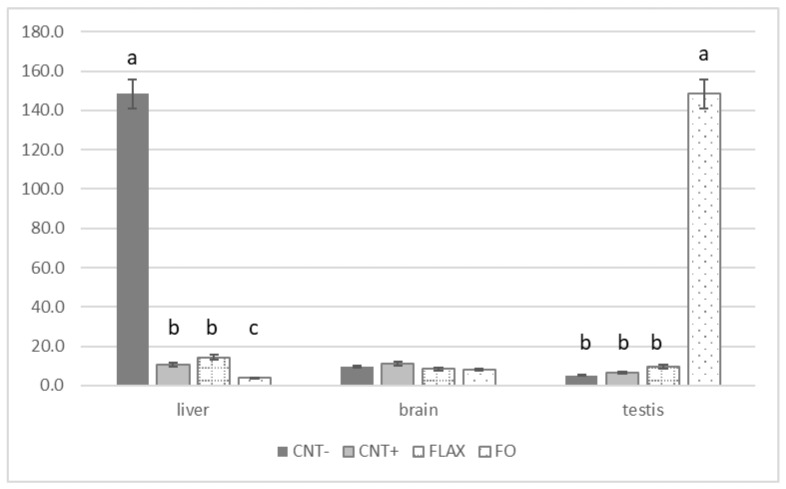
The effect of experimental diets on the gluthatione reduced (GSH) and oxidised (GSSG) ratio, both expressed as nmol/mg protein—in rabbit liver, brain, and testis. The bars represent least squares means + 95% upper and lower limits. The letters a–c indicate a significant difference between dietary groups (*p* < 0.05, Bonferroni post hoc test).

The forms of glutathione (Figure 3) were different in relation to the organs. In the liver, the GSH/GSSG ratio was higher in the CNT−. Conversely, in the testis, the FO group had the highest ratio compared with the other groups. The brain showed no significant differences.

**Figure 4:**
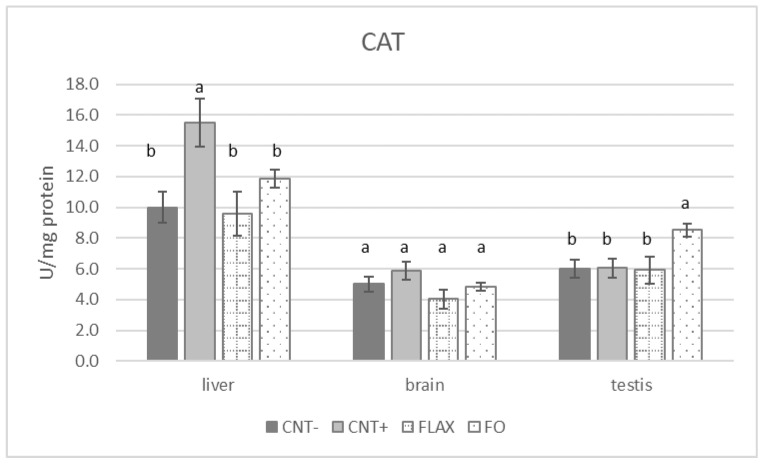
The effect of experimental diets on catalase (CAT) activity in rabbit liver, brain, and testis. The bars represent least squares means + 95% upper and lower limits. a, b indicate a significant difference between dietary groups (*p* < 0.05, Bonferroni post hoc test).

Finally, the CAT activity (Figure 4) was highest in the CNT+ group in the liver (*p* < 0.05), whereas the brain did not show significant differences (*p* > 0.05). In the testis, the CAT activity was highest in the FO group compared with that in all the other groups.

### 3.2. Fatty Acid Profile and Generation of Isoprostanoids

#### 3.2.1. Precursors and Derivatives of n-6 and n-3 Polyunsaturated Fatty Acids

**Figure 5:**
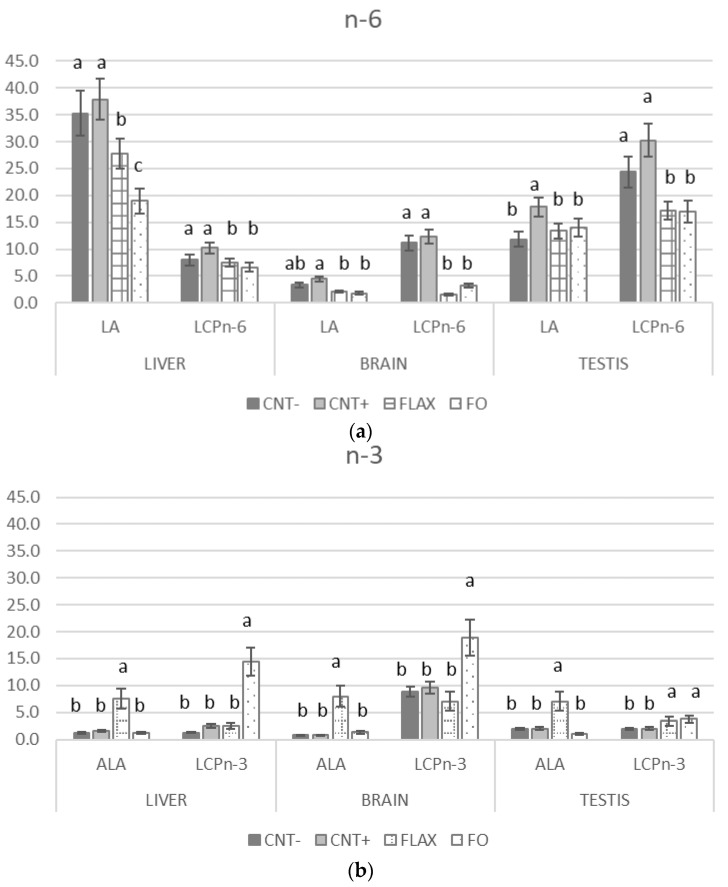
The effect of experimental diets on the long-chain fatty acid (LCP) content of rabbit liver, brain, and testis (presented as % of total fatty acids (FAs)) for the (**a**) n-6 and (**b**) n-3 polyunsaturated fatty acid (PUFA) series. The bars represent least squares means + 95% upper and lower limits. a, b indicate a significant difference between dietary groups (*p* < 0.05, Bonferroni post hoc test).

Figure 5 shows the FA profile (precursor: LA and ALA, derivatives: n-6 and LCPn-3) of different organs. The LA and LCPn-6 levels of the control groups (CNT− and CNT+) were higher than in the n-3 PUFA–supplemented groups in all organs except for the brain, where no statistical significance was recorded for LA. Moreover, the addition of alpha-tocopheryl acetate to the control diet (CNT+) led to a higher n-6 PUFA content compared with that in the CNT− group (Figure 5a). By contrast, the n-3 PUFA content reflected the dietary source (flaxseed of fish oil). In both the liver and brain, the highest proportion of LCPn-3 was in the FO group, whereas the highest level for ALA was in the FLAX group. In the testis, the trend was almost the same as that in the other tissues except that the LCPn-3 level was higher in the FLAX and FO groups with respect to the CNT− and CNT+ groups independently of the n-3 PUFA source.

#### 3.2.2. F_2_-Isoprostane, F_3_-Isoprostane, and F_4_-Neuroprostane Generation

**Figure 6:**
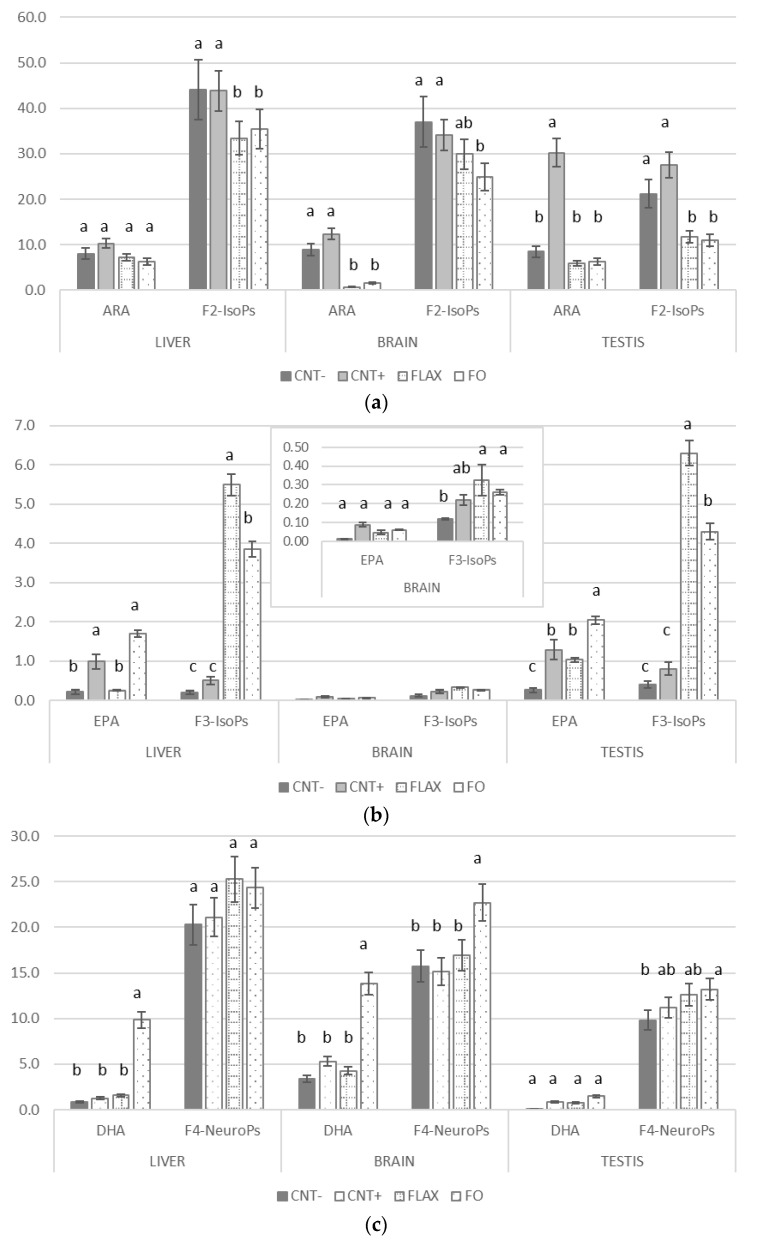
The effect of experimental diets on fatty acid (FA) precursors (arachidonic acid [ARA], eicosapentaenoic acid [EPA], docosahexaenoic acid [DHA]; % of total FA) of isoprostanoids (IsoPs, pg/mL) in rabbit liver, brain, and testis for (**a**) F2-IsoPs, (**b**) F3-isoPs, and (**c**) F4-NeuroPs. When values differences were too wide for showing a complete graph, a support window was added to the main one. The bars represent least squares means + 95% upper and lower limits. a–c indicate a significant difference between dietary groups (*p* < 0.05, Bonferroni post-hoc test).

Figure 6 presents the F_2_-IsoP, F_3_-IsoP, and F_4_-NeuroP concentrations in the different organs, as well as the relative abundance of their respective FA precursors. F_2_-IsoPs (Figure 6a), which originate from ARA, were higher in the CNT− and CNT+ groups of all organs with respect to the FLAX and FO groups. Regarding F_3_-IsoP generation (Figure 6b), the EPA (the precursor) level was higher in the liver and testis of the FO group, whereas there were no differences among the groups in the brain. Furthermore, all the organs showed higher levels of F_3_-IsoPs in the FLAX group compared with that in the CNT− and CNT+ groups. The F_4_-NeuroP concentration (Figure 6c) followed the trend of its precursor (DHA) in the brain, but the DHA level was higher in the FO group compared with that in the other groups. In the testis, the FO group showed slightly higher F_4_-NeuroPs than the CNT− group.

### 3.3. Discriminant Analysis

**Figure 7:**
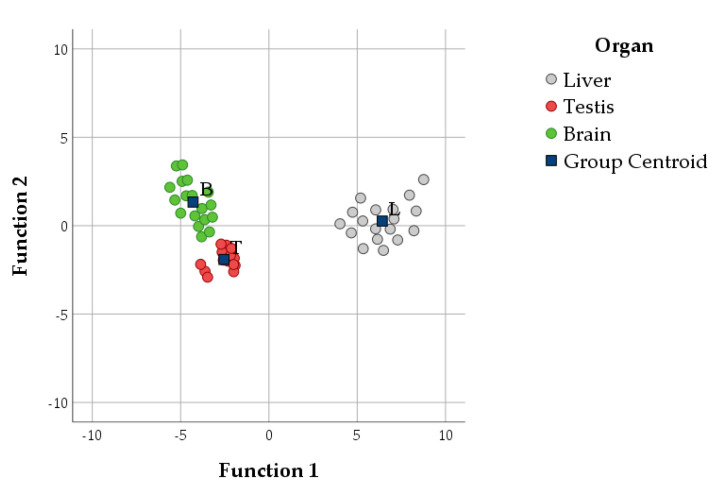
Scatterplot of canonical discrimination analysis (DA) including variables indicating antioxidant features (DA1) with the centroids of each organ (blue squares). L, liver; T, testis; B, brain.

**Figure 8:**
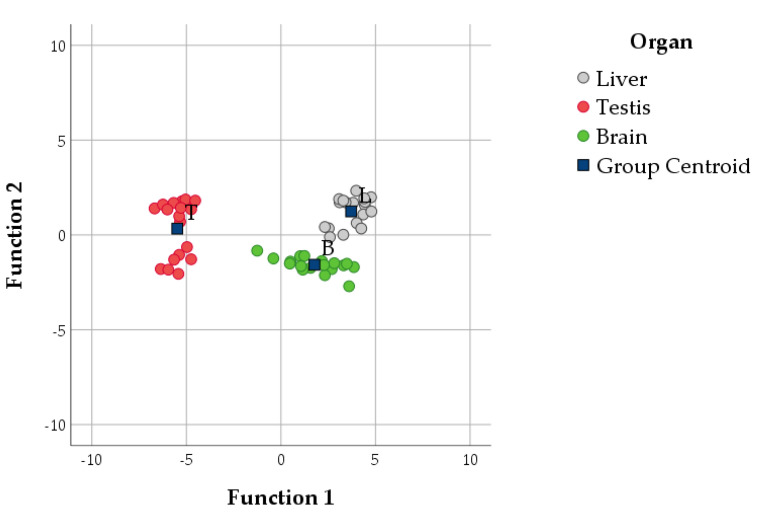
Scatterplot of canonical discrimination analysis (DA) including variables indicating the lipid and isoprostanoid profile (DA2) with the centroids of each organ (blue squares). L, liver; T, testis; B, brain.

In the two DAs (Figure 7 and Figure 8, Table 2), the Mahalanobis distance did not identify any multivariate outlier and all within-groups correlations were <0.8. Two discriminant functions were obtained in each DA. DA1 (Figure 7, Table 3) showed that GPX, vitamin E, and CAT were the variables that mainly contributed to characterise the organs. Indeed, they had the highest loadings in the first function, explaining 93.0% of the variance in the model (Table 3). In particular, centroids indicated that the liver had high scores while the brain and testis had negative scores for this discriminant function (Figure 7, Table 3). Function 2 was mostly explained by GR and AA, but it only contributed to 7.9% of the variance. Function 2 mostly discriminated the brain (positive mean scores) from the testis (negative mean scores).

DA2 (Figure 8, Table 4) showed that F_2_-IsoPs and LCPn-6 were the variables that differed the most according to the organ. They had opposite signs in Function 1, explaining 91.9% of the variance, and mainly discriminated the liver from the testis. In Function 2, F_3_-IsoPs and F_4_-NeuroPs had positive loadings, while LCPn-3 had a negative loading (Table 4). Discriminant scores for function 2 (Figure 8, Table 4) suggested that the liver had slightly higher values for F_3_-IsoPs and F_4_-NeuroPs but low LCPn-3 values; the brain had an opposite pattern.

The two DAs were able to assign most samples correctly to the three organs (96.1% and 98.1% of original grouped cases were correctly classified by DA1 and DA2, respectively).

## 4. Discussion

The oxidative and antioxidant profile was investigated in three rabbit tissues: the liver, chosen as the lipid metabolism centre, the brain as an LCPn-3-sensitive tissue, and the testis as an oxidation-sensitive organ. All these tissues contain a high quantity of PUFAs; however, there is limited data on how their PUFA profile changes due to different dietary inputs.

**Figure 9:**
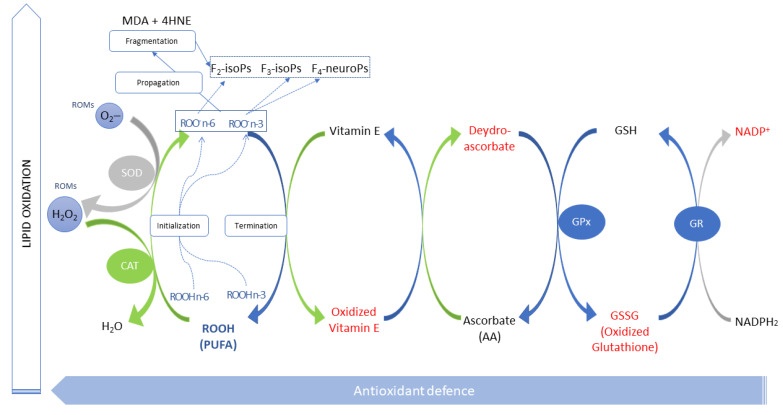
The main steps of the polyunsaturated fatty acid (PUFA) oxidative chain and antioxidant interactions. The red colour indicates oxidised molecules. MDA, malondialdehyde; 4HNE, 4-hydroxynonenal; GSH, glutathione; GPX, glutathione peroxidase, GR, glutathione reductase; F_2-, 3_-isoPs, F_2-_, _3_-isoprostanes; F_4_-NeuroPs, F_4_-neuroprostanes; SOD: superoxide dismutase; CAT: catalase: ROMs: reactive oxygen metabolites.

PUFAs have a high susceptibility to oxidation via free radicals due to their double bonds [55], which constitute attack sites for electrically charged molecules that start the oxidative chain. Because lipid oxidation is involved in many pathological processes, including cancer, neurodegeneration, and diabetes, ways to reduce lipid peroxidation have been widely studied [25]. After the initialisation of the reaction, which is promoted by reactive oxygen metabolites (ROMs, Figure 9), the peroxyl radicals tend to neutralise themselves by reacting with other substances (Propagation, Figure 9), thereby causing a self-perpetuating chain [56] until a final step (Termination, Figure 9) with the generation of secondary products of the oxidative degradation (Fragmentation, Figure 9): i.e., aldehydes, such as MDA and 4-hydroxynonenenal (4HNE), conjugated dienes, and isoprostanoids [57]. Furthermore, the oxidation of some molecules (prostaglandins) originated by the LCP metabolism generates other oxidised forms (i.e., isoprostanoids).

Many antioxidants are involved in the initial and final steps of the oxidative chain. In the first step, some enzymes counteract the effects of ROMs—detoxifying oxygen radicals (i.e., superoxide dismutase (SOD) and CAT [58])—while in the final step the presence of adequate quantities of non-enzymatic antioxidants (i.e., vitamin E and AA) leads to a lower production of secondary metabolites (e.g., MDA). Oxidation largely depends on the number of double bounds of the PUFA; indeed, the free radical–mediated oxidation of FA decreases in the following order: 22:6 (DHA) > 20:4 (ARA) > 18:3 (ALA) > 18:2 (LA) > 18:1 (oleic acid) > 16:0 (palmitic acid) [25].

In the rabbit bucks fed flaxseed, ALA but not LCPn-3 increased in all the analysed organs. A higher LCPn-3 content was recorded in the liver and brain only when fish oil was administered, whereas the testis did not show any significant differences in LCPn-3 with any dietary plan. This trend was probably due to the specific cell membrane composition of the rabbit testis, where LCPn-6 are the main FAs (22:5n-6 was the primary one [1,22]). Furthermore, highly productive rabbit strains show greater conversion of LA into ARA than ALA into LCPn-3 [59]. On the other hand, the dietary administration of n-3 PUFAs reduced LCPn-6 in the testis. In agreement, the trend towards lower DPA from the n-6 PUFA series and higher DHA proportions was also observed in the seminal fluid of bulls [60].

Many reports suggest that cell membrane FAs not only serve as the building blocks of the membrane (phospholipids) and as an energy source (via β-oxidation), but their derivatives also engage in cell signalling [61]. Specifically, oxidised products of LCPs are identified in various body districts and are associated with several metabolic processes and some chronic diseases. These compounds are namely isoprostanes and neuroprostanes, which can be found in low concentrations compared with the FA precursors.

In male infertility, isoprostanes may be considered specific biomarkers of (a) exposure to chemical aetiological agents, (b) oxidative damage, (c) reduced antioxidant response, and (d) sperm immaturity [8]. F_2_-IsoPs are initially formed in situ, esterified into phospholipids, then released by phospholipase [62] and the free form can be considered a specific marker of oxidative stress [63,64].

We found that the F_2_-IsoP levels were higher in the CNT+ and CNT− groups compared with the n-3 PUFA–supplemented groups (FLAX and FO), consistent with the higher concentration of the substrate (ARA). The same was true for the F_3_-IsoPs, which are the products of EPA oxidation: higher dietary administration of n-3 PUFAs resulted in higher F_3_-IsoPs. Of note, the F_4_-NeuroP trend seems to be tissue specific. Although DHA was higher in all body organs (mainly the liver and brain) of the n-3 PUFA–supplemented groups, the F_4_-NeuroP levels were not significantly different in n-3 PUFA–supplemented groups compared with the liver and testis of the CNT− and CNT+ groups, whereas the level was higher in the brain of the FO group. These levels were probably affected by the PUFA composition (precursors of isoprostanes) of the different organs.

The brain is particularly rich in PUFAs such as DHA, and changes in the PUFA composition of its plasma membranes reflect the dietary source. Accordingly, a recent paper suggested that the choice of an oxidation biomarker should be made in relation to the FA composition of the tissues and diets. Specifically, the authors demonstrated that F_4_-NeuroPs increased in the blood and seminal plasma of rabbits fed diets enriched with n-3 PUFAs [22], whereas the F_2_-IsoP quantity was reduced. In humans, F_4_-NeuroPs are considered a marker of structural and functional integrity. Indeed, the decline in integrity of this tissue appears to correlate with the loss of DHA in membranes and the increase in F_4_-NeuroPs, as demonstrated in many neurodegenerative disorders (Parkinson’s disease and Alzheimer’s disease). Our results demonstrate that the dietary intake of PUFAs also affects the F_4_-NeuroP level, suggesting that their variation could also be related to metabolic changes.

Free radical–mediated lipid peroxidation may be inhibited by restraining the initiation and the chain propagation and/or quickening chain termination [25] (Figure 9) by enzymatic or non-enzymatic antioxidants. Cells have a system for the reduction of lipid hydroperoxides; it comprises various antioxidant enzymes with different structures, substrate specificities, and localisations. Accordingly, two main antioxidant systems have been described: (a) removal of ROMs by enzymes such as SOD, CAT, and GPX and (b) scavenging of free radicals by electron donors, such as GSH, vitamin E, AA, carotenoids, flavonoids, amino acids, phospholipids, and thioredoxin, which are antioxidants typically found in foods [65].

In this study, there was a cooperative interaction among antioxidants, and this activity varied in each organ. The testis showed the highest MDA concentration followed by the brain and the liver, with a trend affected by the dietary supplementation (PUFA and/or vitamin E). Besides the dietary PUFA content, the MDA level was also related to the antioxidant content of tissues: indeed, the CNT− group showed higher MDA levels in all organs, such as FO and FLAX groups (Figure 1). The vitamin E concentration followed an inverse trend compared with the MDA concentration and, at the same time, its level in cell membranes appeared to be maintained by high AA levels [66,67]. AA had an important role in restoring oxidised vitamin E: low AA levels were associated with higher vitamin E levels. Moreover, an adequate quantity of AA and vitamin E corresponded to less oxidation mainly in the liver, probably due to the metabolic destiny of this organ. The liver is the master regulator of the vitamin E level in the body; it controls the concentration of the main vitamin E isoform (α-tocopherol) and appears to be the major site of vitamin E metabolism and excretion. Exogenous vitamin E is absorbed and delivered to the liver, and subsequently the α-tocopherol isoform is preferentially recognised by the α-tocopherol transfer protein (α-TTP) and is transferred to the plasma, while the other vitamin E forms (e.g., γ-tocopherol or tocotrienols) are removed from the circulation [68]. The vitamin E levels recorded in the brain and testis were related to the remarkable increase in ALA (testis) and LCPn-3 (brain), which probably determined an exceptional consumption of vitamin E.

As previously stated, other antioxidants interfere with PUFA peroxidation. Indeed, the glutathione enzyme family (GPX and GR) acted in cooperation with vitamin E and AA, in turn restoring AA via the ascorbate-GSH cycle (Figure 9) [69]. GPX is a family of enzymes that require reduced GSH as a substrate. They provide a primary defence against oxidation, using GSH as an electron donor, and are a potent detoxifier of xenobiotics and represent a barrier against hydroperoxide-induced oxidation [70]. CAT is present in most tissues and is considered a major antioxidant enzyme, catalysing the decomposition of H_2_O_2_ to H_2_O and O_2_ [71]. CAT removes H_2_O_2_ peroxidically and, thus, reduces the initialisation step of oxidation.

As demonstrated from the trend of the different traits considered in this study, independently of the diets administered, each tissue clustered separately and was affected by different antioxidant and lipid pathways (Figure 7 and Figure 8), as described below.

As the centre of lipid metabolism, the liver displayed a strong oxidant/antioxidant pathway chain, mainly modulated by the GPX activity and secondarily by vitamin E and CAT. Furthermore, the main lipid pathway seemed to be isoprostanoid generation, mainly constituted by F_2_-IsoPs.

In the brain, LCPn-3 had a greater, although mild, influence; this is expected because this organ is the main n-3 PUFA–sensitive tissue. However, the brain showed low isoprostanoid production. The antioxidant pathway was mainly characterised by the activity of secondary antioxidant molecules—i.e., GR and AA—which functioned to restore key molecules (e.g., vitamin E).

In the testis, the antioxidant pathway had a relatively small influence, probably due to the establishment of a balance between oxidative and antioxidant response [1]. Regarding the lipid profile, the testis showed a propensity for LCPn-6 accumulation and low isoprostanoid generation.

## 5. Conclusions

The addition of dietary PUFA affects the oxidative status of several organs. In particular, the level of different isoprostanoids can be considered valid biomarkers, besides the evaluation of MDA, to estimate the specific oxidative status of tissues. We delineated a chain mechanism of antioxidant molecules in the different organs; it largely depends on the PUFA composition and the biological fate of the tissues. We confirmed that the liver is the primary metabolic site because both antioxidant and lipid pathways are widely modulated by PUFA and vitamin E administration. On the other hand, the brain seemed to be particularly affected by the dietary lipid source, whereas the testis was affected by the oxidant/antioxidant pathway. Nevertheless, further investigations are needed to understand the respective role of various isoprostanoids in the different organs (F_2_-IsoPs, F_3_-IsoPs, and F_4_-NeuroPs), also considering that every organ showed a unique trend, associated with the FA profile and their interaction with the antioxidants contained in each organ.

## Figures and Tables

**Table 1:** Formulation and proximate analysis of the control and n-3 polyunsaturated fatty acid (PUFA)-enriched diets.

Ingredients	Units	CNT−	CNT+	FLAX	FO
Dehydrated alfalfa meal	g/kg	300	300	380	380
Soybean meal 44%	g/kg	150	150	100	150
Barley meal	g/kg	410	410	310	335
Wheat bran	g/kg	52	52	52	52
Soybean oil	g/kg	30	30	-	-
Extruded flaxseed	g/kg	-	-	100	-
Fish oil ^1^	g/kg	-	-	-	35
Beet molasses	g/kg	20	20	10	10
Calcium carbonate	g/kg	7	7	7	7
Calcium diphosphate	g/kg	13.5	13.5	13.5	13.5
Salt	g/kg	7	7	7	7
DL-methionine	g/kg	0.5	0.5	0.5	0.5
Vitamin–mineral premix ^2^	g/kg	10	10	10	10
Alpha-tocopheryl acetate	mg/kg	-	150	150	150
Crude protein	g/kg	175	174	174	175
Ether extract	g/kg	480	477	472	425
Crude fibre	g/kg	124	122	137	130
Ash	g/kg	89	89	84	90

^1^ NORDIC NATURALS omega-3^®^ is purified deep sea fish oil (from anchovies and sardines) containing eicosapentaenoic acid (EPA) acid 330 mg/100 g, docosahexaenoic acid (DHA) 220 mg/100 g, other long-chain polyunsaturated fatty acid derivatives (LCPn-3) 140 mg/100 g. ^2^ Per kg diet: vitamin A 11,000 IU; vitamin D3 2000 IU; vitamin B1 2.5 mg; vitamin B2 4 mg; vitamin B6 1.25 mg; vitamin B12 0.01 mg; alpha-tocopheryl acetate 5 mg; biotin 0.06 mg; vitamin K 2.5 mg; niacin 15 mg; folic acid 0.30 mg; d-pantothenic acid 10 mg; choline 600 mg; Mn 60 mg; Fe 50 mg; Zn 15 mg; I 0.5 mg; Co 0.5 mg.-Indicates that the specific ingredient was not included in the diet.

**Table 2:** Functions at group centroids (mean discriminant scores for each organ) of discriminant analysis 1 (DA1, Figure 7) and DA2 (Figure 8) describing antioxidants and the lipid profile, respectively.

Organ.	DA1		DA2	
	Function 1	Function 2	Function 1	Function 2
Liver	6.418	0.260	3.713	1.241
Testis	−2.546	−1.913	−5.483	0.332
Brain	−4.296	1.334	1.770	−1.574

**Table 3:** Discriminant loadings of variables indicating antioxidants included in discriminant analysis 1 (DA1) with percentage of variance, Wilks’s lambda, and the significance of each discriminant function.

	Function	
	1	2
GPX	0.567 *	0.441
Vitamin E	0.340 *	−0.217
Catalase	0.326 *	−0.177
GR	−0.109	0.427 *
AA	−0.096	0.326 *
Percentage of variance	93.0%	7.0%
Wilks’ lambda	0.014	0.353
Significance (*p*)	<0.001	<0.001

The variables are ordered by the absolute size of the correlation within the function. * Indicates the largest absolute correlation between each variable and any discriminant function; 96.1% of original grouped cases were correctly classified. AA, ascorbic acid; GPX, glutathione peroxidase; GR, glutathione reductase.

**Table 4:** Discriminant loadings of variables indicating lipids and isoprostanoids included in discriminant analysis 2 (DA2) with percentage of variance, Wilks’s lambda, and the significance of each discriminant function.

	Function	
	1	2
F_2_-IsoPs	0.445 *	−0.373
LCPn-6	−0.199 *	−0.103
F_3_-IsoPs	−0.089	0.544 *
F_4_-NeuroPs	0.418	0.510 *
LCPn-3	0.119	−0.273 *
Percentage of variance	91.9%	8.1%
Wilks’ lambda	0.023	0.407
Significance	<0.001	<0.001

The variables are ordered by the absolute size of the correlation within the function. * Indicates the largest absolute correlation between each variable and any discriminant function. IsoP, isoprostane; LCP, long-chain fatty acid; NeuroP, neuroprostane.

## Data Availability

Data available on request from the authors.

## Abbreviations

AA, ascorbic acid; ALA, alpha-linolenic acid; ARA, arachidonic acid; CAT, catalase; DA, discriminant analysis; DHA, docosahexaenoic acid; EPA, eicosapentaenoic acid; F_2_-IsoPs, F_2_-isoprostanes; F_3_-IsoPs, F_3_-isoprostanes; F_4_-NeuroPs, F_4_-neuroprostanes; FA, fatty acid; FAME, fatty acid methyl ester; GPX, glutathione peroxidase; GR, glutathione reductase; GSH, glutathione reduced; GSSG, glutathione oxidised; LA, linolenic acid; LCP, long-chain fatty acid; MDA, malondialdehyde; MUFA, monounsaturated fatty acid; PGF_2α_, prostaglandin F_2α_; PUFA, polyunsaturated fatty acid; ROMs, reactive oxygen metabolites; SFA, saturated fatty acid; Vit E, vitamin E.
